# Mammographically Occult Invasive Lobular Carcinoma With Intradermal Invasion

**DOI:** 10.7759/cureus.27358

**Published:** 2022-07-27

**Authors:** Shefali Khanna, Yana Puckett

**Affiliations:** 1 Department of Surgery, Charleston Area Medical Center, Charleston, USA

**Keywords:** inflammatory breast cancer, occult carcinoma, diagnostic mammography, breast cancer detection, invasive lobular breast carcinoma

## Abstract

While shortcomings in the detection of invasive lobular carcinoma (ILC) continue to be studied, research is ongoing to determine detection rates using current breast imaging modalities in combination with physical examination findings. In the following case report, we describe the rare presentation of a patient diagnosed by punch biopsy with grade III, estrogen receptor (ER)-/progesterone receptor (PR)-positive invasive lobular carcinoma with intradermal invasion. This patient presented with findings similar to inflammatory breast cancer (IBC) including pain in the left nipple, skin warmth, and erythema circumferentially encompassing approximately two-thirds of the left breast. This case study is of significance as, to date, it is the first report of an invasive lobular carcinoma that presented clinically as inflammatory breast cancer and was occult on both diagnostic mammography and ultrasound. While imaging remains the primary method of breast cancer detection, it is important to note that clinical findings of dermal invasion of the breast may prompt further investigation with a biopsy and close follow-up, regardless of imaging results.

## Introduction

The most recent breast cancer statistics divulge that approximately one out of every eight females in the United States will be diagnosed with invasive breast cancer, although this is slowly downtrending. Invasive lobular carcinoma (ILC) is the malignancy of the breast that begins in lobules or milk-producing glands [1]. It is the second most common subtype of invasive breast cancer behind invasive ductal carcinoma (IDC), which is a malignancy located in breast ducts. ILCs account for roughly 5%-15% [2] of new breast cancers diagnosed and affect roughly 10% of people diagnosed specifically with invasive breast cancer [3]. In this case report, we describe a patient with invasive lobular carcinoma with an intradermal invasion that was mammographically and ultrasound occult.

## Case presentation

A 73-year-old female with a past medical history of hypertension, gastroesophageal reflux disease, and anxiety was referred to our physicians from an outlying facility, where she followed with a general surgeon and was diagnosed with invasive lobular carcinoma of the left breast after punch biopsy. Prior to seeing a general surgeon, the patient was treated for mastitis of the left breast for a total of eight months with antibiotics without success. A punch biopsy of the skin changes was performed in the clinic setting with pathology revealing a grade III, estrogen receptor (ER)-positive (99%), progesterone receptor (PR)-positive (100%), HER2-negative invasive lobular carcinoma. No comment on dermal invasion was made. She was placed on tamoxifen.

**Figure 1:**
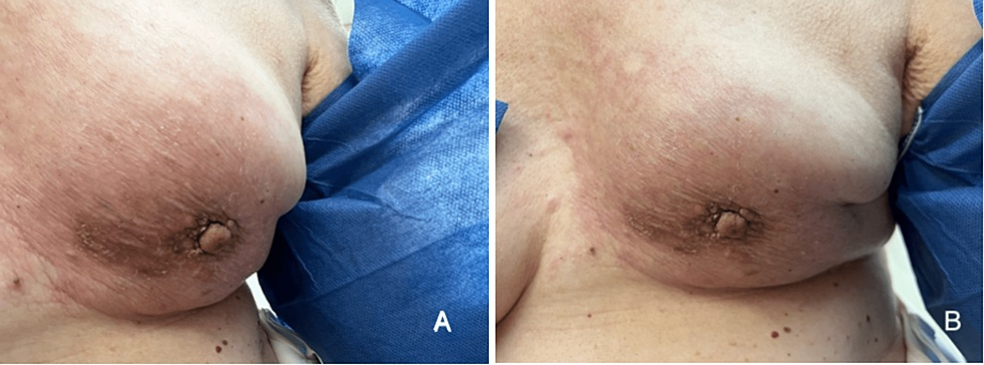
Physical examination findings. (A and B) Erythema encompassing approximately two-thirds of the left breast circumferentially with mild ulceration alongside skin dimpling and retraction.

**Figure 2:**
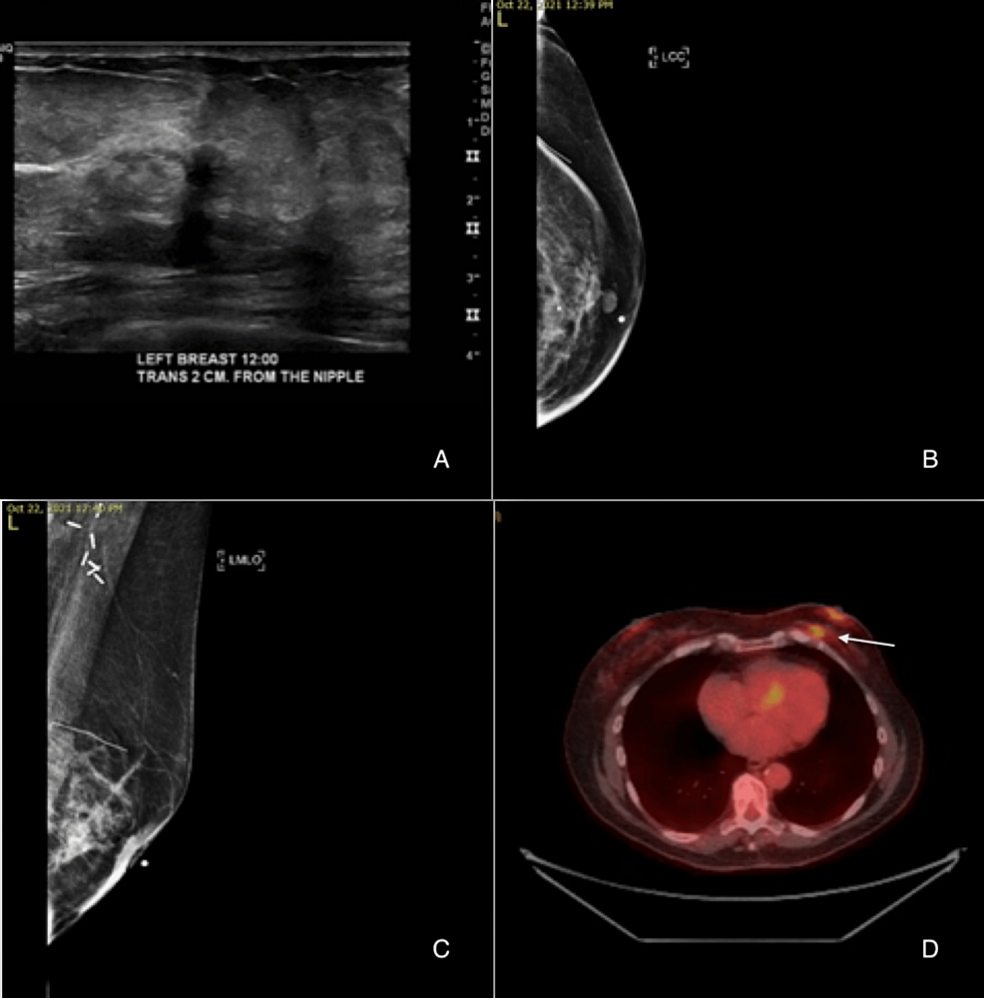
Imaging results. (A) Negative breast ultrasound. (B) Negative left breast mammogram in the mediolateral oblique view. (C) Negative left breast mammogram in the craniocaudal view. (D) PET scan findings displaying a 2-cm lesion in the left breast (arrow).

The patient had a history of lumpectomy 21 years ago of the same breast, followed by four cycles of chemotherapy and 35 sessions of adjuvant radiation for invasive ductal carcinoma with receptor status unknown. She reported pain in the left nipple with skin warmth and swelling of the affected breast but denied weight loss, pallor, nausea, bone pain, cough, and night sweats upon presentation. A physical examination revealed erythema that encompassed approximately two-thirds of the left breast circumferentially with mild ulceration alongside skin dimpling and retraction as noted in Figure 1. There was a dominant palpable mass roughly 2 cm in size just behind the nipple-areolar complex. No palpable lymph nodes were detected. Imaging with diagnostic tomosynthesis mammogram and ultrasound failed to reveal a mass in the left breast. However, a PET-CT scan showed a 2-cm lesion in the left breast that seemed to be intradermal (Figure 2).

An MRI of the bilateral breasts revealed a cyst in the medial portion of the right breast, estimated at approximately 5 mm × 4 mm in size. In the left breast, intradermal tissue thickening was noted that was questioned to possibly be postsurgical scarring. Due to the complexity of this patient’s case, after a multidisciplinary tumor board presentation, it was recommended that the patient be treated as inflammatory breast cancer (IBC) given the prior history of carcinoma in the same breast necessitating lumpectomy and chemoradiation 21 years ago. Protocol per Giordano and Hortobagyi in breast cancer research was utilized [4]. The patient underwent neoadjuvant chemotherapy with docetaxel 75 mg/m^2^ IV every 21 days for four cycles and Cytoxan 600 mg/m^2^ IV every 21 days for four cycles.

**Figure 3:**
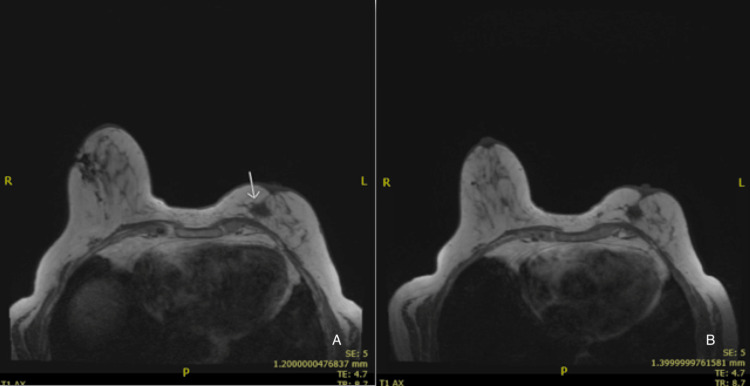
MRI findings. (A) Pre-chemotherapy. (B) Post-chemotherapy.

MRI of the breasts post-neoadjuvant chemotherapy revealed no abnormal enhancement bilaterally. The right breast was read as unremarkable, and the same intradermal tissue thickening of the left breast mentioned above was appreciated once again (Figure 3). There did not appear to be much change. Given this, surgical management was discussed with the patient, and she underwent left breast modified radical mastectomy. Postoperatively, pathology revealed invasive lobular carcinoma, and she completed a full course of adjuvant radiation therapy. She progressed very well, carrying out all activities of daily living, and has been scheduled for a routine screening mammogram later this year.

## Discussion

Often, the initial and only prevailing finding of invasive lobular carcinoma can be thickness and excess firmness of the breast, in contrast to the usual bump reported by patients as an early representation of invasive ductal carcinoma. Other reported symptoms of ILC are breast pain or pressure, inversion of the nipple, texture changes of the skin, a feeling of fulness/heaviness in the breast, swelling, or dimpling [5]. In our patient, the physical examination findings mimicked inflammatory breast cancer (IBC). This sort of presentation does not often occur, and a study of females with IBC performed by Raghav et al. revealed that 4.6% of patients in the study were identified with ILC. In addition, lobular histology was visualized in just 8% of invasive breast carcinoma [6].

In regard to diagnostic workup, it has long been known that the detection of this cancer with imaging has posed limitations, and research is ongoing about the incidence rates of mammographically occult ILC. Studies have revealed that mammography displays between a 57% and 79% sensitivity for showing ILC. Johnson et al. reported that up to 30% of ILC cases are not apparent on mammography, and 35% of ILCs are only detectable in one view. The abnormal cells’ low density and their lack of desmoplastic stromal reaction are partially attributed to these findings, as well as the lack of recognition with a physical examination. These can also play a role in the lack of detection even on pathologic evaluation [2].

Literature shows that compared to other kinds of invasive carcinoma, ILC is more likely to affect both breasts [7]. Our patient’s skin findings appeared to slightly begin spreading to the contralateral breast; however, a biopsy of the opposite breast in our patient was negative for contralateral spread. A recent estimate was that for females who have LCIS, their chances of going on to acquire invasive carcinoma were between 30% and 40%, but the progression is not at all rapid, generally occurring in greater than 15 years. The overall prognosis of ILC remains very favorable as most diagnoses are usually low grade. Generally, a majority of lobular cancers are found to be hormone receptor-positive (typically ER-positive) and are quite responsive to hormone treatment [5]. Since 1975, the five-year survival rate has steadily improved [8]. A study published in the British Journal of Cancer revealed that the incidence of females with ILC who had axillary nodal metastasis was less than in their female counterparts with IDC (43% versus 53%, P = 0.02). Of note, a difference in the primary tumor size was not found between these two groups. Compared to IDCs, ILCs were discovered to more often be of lower grade, display lower mitotic counts, and cause less tumor necrosis. Small pT1NOMO ILCs (n = 41) had 100% 10-year and 83% 20-year corrected rates of survival [9].

Invasive micropapillary carcinoma (IMPC) is a rare histological subtype of carcinoma that is associated with its difficulty in accurate imaging estimation. It is often associated with larger tumors and a higher percentage of positive lymph nodes due to well-recognized lymphovascular tropism [10]. Given the patient’s palpable 2-cm lesion and lack of positive lymph nodes, this was not considered. In addition, the patient discussed in this case report had an intradermal invasion of her cancer as noted above. A recent case report by Eaton et al. described two cases of women with dermal involvement of their ILC coincidentally observed on imaging [11]. With this in consideration, dermal extension or lesions can be seen on MRI; however, significance can often be unclear. Often, skin lesions are of little consequence, such as benign cysts. However, in conjunction with a mass and cancer-like symptoms or in the setting of known current or previous cancer, minimal invasion and spread should never fail to be taken into consideration [12]. The best way to view dermal developments or suspicion of dermal involvement is with sonography, especially through the utilization of a high-frequency transducer. It is incredibly valuable to consider malignancy with intradermal lesion findings of the breast on ultrasound. Although the answer may never be confirmed, we question whether this was a true inflammatory cancer versus a locally advanced cancer due to its nontraditional presentation.

## Conclusions

As in the presented case, invasive lobular carcinoma is well known to generally be discrete on imaging, and the challenges in its detection are an ongoing topic of debate and study. We recommend that due to the fact that intradermal invasion can emerge with findings that may initially appear benign, such as dermal findings in this patient, it is of paramount importance to investigate these findings with further imaging or biopsy. Furthermore, we believe radiologists should be involved to thoroughly assess any such finding further if clarification is needed, and when necessary, ultrasound, mammogram, clinician physical examination, and dermatology or additional consultations can be invoked to aid with a definitive diagnosis. On the same token, the importance of adequate follow-up cannot be understated. To date, this is the only case report that presents an ILC that was both mammographically and ultrasound occult that presented clinically as inflammatory breast cancer.
